# Surgical Intervention for Filariasis-Induced Lymphedema: A Systematic Review of Ablative and Physiological Approaches

**DOI:** 10.3390/life16060942

**Published:** 2026-06-02

**Authors:** Rani Septrina, Irra Rubianti Widarda, Putie Hapsari, Valeska Siulinda Candrawinata

**Affiliations:** 1 Division of Plastic and Reconstructive Surgery, Department of Surgery, Faculty of Medicine, Universitas Padjadjaran/Dr. Hasan Sadikin General Hospital, Bandung 40161, Indonesia; irrarw@gmail.com; 2 Division of Vascular and Endovascular Surgery, Department of Surgery, Faculty of Medicine, Universitas Padjadjaran/Dr. Hasan Sadikin General Hospital, Bandung 40161, Indonesia; putie.hapsari@unpad.ac.id; 3 Department of Surgery, Universitas Padjadjaran/Dr. Hasan Sadikin General Hospital, Bandung 40161, Indonesia; valeska24001@mail.unpad.ac.id

**Keywords:** elephantiasis, filariasis, lymphedema, surgery

## Abstract

Advanced filariasis-induced lymphedema causes irreversible fibrosis, severe disability, recurrent infections, and major psychosocial burden, often with poor response to conservative therapy. Surgical management in this type of lymphedema is rarely addressed. We conducted a PRISMA 2020-guided systematic review evaluating surgical interventions versus conservative management or no intervention in adults with filarial lymphedema, involving various anatomical locations. Of 580 records identified, 29 studies were included for qualitative synthesis. The evidence base predominantly consisted of case reports, with surgical indications frequently involving disabling elephantiasis or diagnostic excision of filarial masses. While ablative procedures (excisional debulking) remained the primary salvage strategy, specific studies documented physiological techniques, including nodovenous shunts and vascularized lymph node transfer (VLNT). Quantitative limb-volume reduction was sparsely reported; however, qualitative assessments demonstrated significant improvements in ambulation, cosmesis, and patient-reported quality of life. Postoperative complications, primarily superficial surgical site infections, were generally low and manageable. Ablative surgical procedures appear to be the most common salvage strategy for advanced filarial lymphedema, but the evidence base remains limited to low-level, heterogeneous observational data. However, with the emergence of physiological lymphatic surgery, a combination of ablative procedures and physiological procedures should be considered. Prospective controlled studies with standardized outcomes are urgently needed.

## 1. Introduction

Lymphatic filariasis remains a leading cause of permanent and long-term disability worldwide [1]. According to the World Health Organization, while millions are infected globally, approximately 36 million people are visibly disfigured and incapacitated by chronic complications such as lymphedema and elephantiasis [2]. The disease progressively damages the lymphatic system, and in its advanced stages, it presents a severe clinical challenge characterized by irreversible fibrosis in the subcutaneous tissue that results in massive limb enlargement and highly debilitating recurrent secondary infections (acute attacks) [3]. For adult patients, particularly those residing in endemic areas, these physical manifestations not only severely impair mobility but also inflict profound psychological and socioeconomic burdens, underscoring the critical need for effective, durable interventions [4].

The traditional cornerstone of care for early-stage filarial lymphedema relies heavily on conservative management, including strict hygiene protocols, compression therapy (bandaging), and pharmacologic regimens such as doxycycline or antifilarials [5]. However, adults presenting with advanced filariasis lymphedema frequently become unresponsive to these non-operative measures, as conservative care is generally insufficient to reverse established fibrotic tissue or adequately halt recurrent infections [6,7]. Consequently, surgical intervention has emerged as a vital salvage strategy. Contemporary surgical approaches range from physiological procedures aimed at restoring lymphatic flow to ablative techniques, such as liposuction and excisional debulking of fibrotic tissue [8]. The choice of physiological surgery, such as lymphovenous anastomosis (LVA) or vascularized lymph node transfer (VLNT), is rarely done in advanced stage of filariasis lymphedema due to less satisfactory results [5].

These surgical approaches have demonstrated growing clinical interest and highly promising outcomes in cases of primary lymphedema, such as 20% to 50% reductions in limb volume, along with improved cosmesis and quality of life in 70% to 90% of advanced cases at 1–2 years of follow-up, the overall comparative efficacy of these techniques remains fragmented [9,10]. The current literature varies widely in surgical approach, comparator groups, and follow-up metrics. Therefore, this systematic review aims to comprehensively evaluate the efficacy of surgical interventions compared to conservative management or no intervention in adults with filarial lymphedema.

## 2. Materials and Methods

Protocol and Guidelines

This systematic review was formulated and executed in strict accordance with the Preferred Reporting Items for Systematic Reviews and Meta-Analyses (PRISMA) guideline 2020 [11]. To ensure a rigorous and reproducible approach to synthesizing the available clinical evidence and to maintain methodological transparency, this study is registered in the PROSPERO system (ID: CRD420261343996) and can be accessed at: https://www.crd.york.ac.uk/PROSPERO/view/CRD420261343996 (registered at 25 March 2026).

Eligibility Criteria

Study inclusion was prospectively defined utilizing the Population, Intervention, Comparison, and Outcomes (PICO) framework. The target population was restricted to adult patients, predominantly situated in endemic regions, who presented with filarial lymphedema. It should be noted that individual studies may have used alternative classification systems, such as the International Society of Lymphology (ISL) staging system, and such discrepancies are indicated in the corresponding table entries. Eligible clinical profiles required the presence of irreversible fibrosis, elephantiasis, recurrent secondary infections, and significant mobility impairment that had proven refractory to standard conservative management strategies, such as Complete Decongestive Therapy (CDT) and antifilarial pharmacotherapy. The interventions under investigation encompassed a spectrum of surgical modalities, ablative surgical techniques, including excisional debulking of fibrotic tissue and liposuction, as well as physiological approaches such as lymphovenous anastomosis (LVA) and vascularized lymph node transfer (VLNT). Studies exploring the combined application of these techniques, such as vascularized lymph node transfer paired with excision, were also deemed eligible.

For comparative purposes, acceptable control groups included patients undergoing conservative management, encompassing strict hygiene protocols, routine bandaging, and doxycycline administration, or those receiving no intervention. The primary outcome measure was the quantifiable reduction in limb volume. Secondary clinical outcomes of interest included changes in the frequency of acute attacks, postoperative cosmesis, and patient-reported quality of life. Methodologically, the review incorporated a broad range of primary study designs, explicitly accepting randomized controlled trials, prospective cohort studies, case–control studies, case series, and case reports. Conversely, the literature was excluded if the studies were preclinical, based on non-human or in vitro models, or categorized as review articles. Furthermore, logistical constraints necessitated that inclusion be strictly limited to English-language publications that were either fully open access or retrievable through our university institutional accounts. Although the target population was adults, certain studies containing mixed-age cohorts, including a minority of pediatric cases, were retained when they contributed relevant surgical data that could not be disaggregated; this deviation is acknowledged as a limitation.

Information Sources and Search Strategy

A comprehensive systematic search was conducted across three primary academic databases: PubMed, EMBASE, and Scopus. The search parameters encompassed all available literature from the respective inceptions of these databases through to 31 January 2026. The search strategy was meticulously developed by synthesizing controlled vocabulary, including MeSH terms, with free-text keywords centralized around the core concepts of lymphedema, filariasis, and surgical interventions. To achieve literature saturation and mitigate the risk of omitting highly relevant data, a supplementary manual screening of the reference lists from all formally included studies and pertinent review articles was also performed.

Risk of Bias Assessment

To ensure the methodological rigor and validity of the synthesized evidence, a comprehensive risk of bias assessment was conducted for all included studies. The Joanna Briggs Institute (JBI) critical appraisal tools were selected for the evaluation of case reports and case series due to their validated applicability for these specific study designs, while the Newcastle-Ottawa Scale (NOS) was applied to the observational cross-sectional study as recommended by the Cochrane Collaboration for non-randomized studies. Two authors independently applied these standardized tools to evaluate each study’s methodological quality, resolving any discrepancies through mutual agreement. This rigorous, independent review approach guaranteed that potential biases related to patient selection, outcome ascertainment, and data reporting were systematically identified.

A meta-analysis was not feasible due to the extreme heterogeneity in study designs, outcome measures, anatomical sites, and follow-up durations across the included studies. Consequently, a qualitative narrative synthesis was determined to be the only appropriate analytical approach for this dataset.

Study Selection and Data Extraction

Following the application of the search algorithms across all databases, the aggregated citations were subjected to a deduplication process. The initial phase of study selection involved a rigorous screening of titles and abstracts to evaluate their alignment with the predefined eligibility criteria. Articles that satisfied these parameters, as well as those providing insufficient data in the abstract for a definitive exclusion, were subsequently advanced to a full-text review. Upon finalization of the included literature cohort, relevant data pertaining to study design, demographic characteristics, specific surgical interventions utilized, and the predefined clinical outcomes were systematically extracted to facilitate the final qualitative synthesis.

## 3. Results

**Figure 1:**
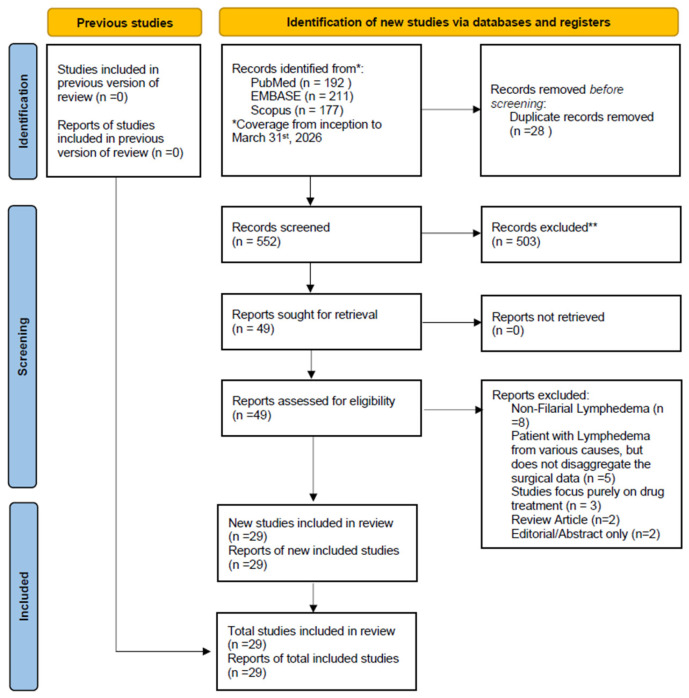
PRISMA flow diagram. * Automation tools were not used for screening. ** No additional records were identified from reference list screening.

The study selection process is illustrated in Figure 1. A total of 580 records were identified from PubMed (*n* = 192), EMBASE (*n* = 211), and Scopus (*n* = 177); after removal of duplicate records (*n* = 28), 552 records were screened, of which 503 were excluded. Full-text reports were sought for 49 records, and all 49 were retrieved and assessed for eligibility (0 not retrieved). Of these, 20 reports were excluded for the following reasons: non-filarial lymphedema (*n* = 8), inclusion of patients with lymphedema from various causes without disaggregating surgical data (*n* = 5), studies focused purely on drug treatments (*n* = 3), review article (*n* = 2), and editorial/abstract-only publications (*n* = 2), leaving 29 studies included in the review (Figure 1) [12,13,14,15,16,17,18,19,20,21,22,23,24,25,26,27,28,29,30,31,32,33,34,35,36,37,38,39,40].

### 3.1. Study Characteristics and Patient Demographics

A total of twenty-nine studies met the eligibility criteria and were included in the final qualitative synthesis (Table 1). The extracted literature predominantly comprised single-patient case reports, alongside one case series, one retrospective database analysis, and one large-scale cross-sectional epidemiological study [12,13,14,15,16,17,18,19,20,21,22,23,24,25,26,27,28,29,30,31,32,33,34,35,36,37,38,39,40]. Geographically, the data reflect the endemic nature of lymphatic filariasis, with a significant majority of the studies originating from India. Additional cases were reported from diverse global locations, including the United States, Thailand, the United Kingdom, Spain, Canada, Denmark, Indonesia, Tanzania, and Pakistan. Excluding the large epidemiological study and the retrospective database analysis, the individual clinical cases represented a diverse age demographic ranging from eleven to seventy-three years [12,13,14,15,16,17,18,19,20,21,22,23,24,25,26,27,28,29,30,31,32,33,34,35,36,37,38,39,40].

Sex distribution was heavily dependent on the specific clinical manifestation, with male patients exclusively represented cases of genitourinary involvement, while female patients predominantly presented with breast-related filarial nodules or cervical masses. Where reported, the duration of clinical symptoms prior to surgical intervention varied drastically, ranging from six months to thirty years. While the initial inclusion criteria targeted advanced lymphedema, documentation of baseline lymphedema staging was notably sparse across the included studies, with only a fraction explicitly confirming grade three or grade four disease [12,13,14,15,16,17,18,19,20,21,22,23,24,25,26,27,28,29,30,31,32,33,34,35,36,37,38,39,40]. Prior conservative management was similarly underreported, though several cases noted the utilization of oral antibiotics, regular wound dressings, and targeted filariasis treatments before surgical escalation [12,13,14,15,16,17,18,19,20,21,22,23,24,25,26,27,28,29,30,31,32,33,34,35,36,37,38,39,40].

### 3.2. Anatomical Locations, Surgical Indications and Interventions

The anatomical distribution of filarial lymphedema was heterogeneous, although the lower extremities and genitourinary regions represented the most common sites of involvement. Lower extremity disease was reported in at least 9 studies and was frequently associated with advanced WHO Grade 3–4 lymphedema, chronic ulceration, impaired ambulation, and recurrent infections. Genitourinary involvement, including penoscrotal and scrotal lymphedema, was reported in 9 studies and often occurred after prolonged disease duration ranging from 3 to 32 years. Less frequent anatomical sites included the facial/periorbital region (2 studies), cervical lymph nodes (1 study), breast (3 studies), upper extremity involvement (3 studies), retroperitoneal or adrenal involvement (3 studies), and hepatic/systemic disease (1 study) [12,13,14,15,16,17,18,19,20,21,22,23,24,25,26,27,28,29,30,31,32,33,34,35,36,37,38,39,40].

The clinical indications prompting surgical intervention across the included cohort were highly heterogeneous (Table 2) and frequently diverged from classical extremity lymphedema management. A substantial proportion of surgeries were indicated for diagnostic uncertainty, such as the evaluation of suspected breast neoplasms, cervical lymphadenopathy, and unexplained facial or retroperitoneal cysts, which were subsequently identified as filarial in origin. Another major surgical indication was the presence of massive, disabling genitourinary elephantiasis, often complicated by secondary infections, chronic ulcerations, or the development of superimposed squamous cell carcinoma.

Among the 29 included studies, excisional and debulking procedures were the predominant surgical approaches, reported in 19 studies. These included subtotal scrotectomy, reduction scrotoplasty, modified Charles procedure, wide local excision, excisional biopsy, cyst excision, and reconstructive procedures involving skin grafts or flap reconstruction. Physiologic or microsurgical lymphatic procedures were less common, reported in 3 studies, and included nodovenous shunt surgery, nodo-venal shunt procedures, and vascularized lymph node transfer (VLNT). Minimally invasive or diagnostic procedures, including fine-needle aspiration cytology (FNAC), fine-needle aspiration (FNA), ultrasound-guided core needle biopsy, nephrostomy, and ureteric stenting, were reported in 5 studies. One study described conservative non-surgical management alone, while several epidemiologic or descriptive studies did not report a definitive operative intervention [12,13,14,15,16,17,18,19,20,21,22,23,24,25,26,27,28,29,30,31,32,33,34,35,36,37,38,39,40].

### 3.3. Clinical Outcomes and Postoperative Complications

An analysis of the defined primary and secondary outcomes revealed significant gaps in the current literature. Most notably, the primary outcome of percentage limb volume reduction was not quantified or reported in any of the included studies, largely due to the anatomical focus of the surgeries shifting toward genitourinary reconstruction and localized nodule excision rather than whole-limb debulking [12,13,14,15,16,17,18,19,20,21,22,23,24,25,26,27,28,29,30,31,32,33,34,35,36,37,38,39,40]. However, qualitative assessments of secondary outcomes demonstrated that surgical intervention generally yielded favorable symptomatic and psychosocial results. Patients undergoing massive excisional debulking for scrotal elephantiasis consistently reported significant relief from physical discomfort, restored ambulation, and highly acceptable cosmetic outcomes that fundamentally improved their quality of life [31,34]. In cases where surgery was performed for localized filarial cysts or nodules, complete symptomatic resolution and disease-free margins were frequently achieved within weeks of the procedure [13,14,15,16,17,19,20,21,22,23,24,25,26,27,28,29,30,31,32,33,34,35,36,37,38,39,40].

The postoperative follow-up durations varied widely, ranging from two weeks to ongoing monitoring over several months, with one database analysis noting significant loss to follow-up. Postoperative outcomes generally demonstrated significant functional and symptomatic improvement across intervention types. Excisional and reconstructive procedures for genital lymphedema consistently resulted in improved mobility, hygiene, cosmesis, psychosocial well-being, and quality of life, with several reports describing minimal recurrence and satisfactory wound healing. Lower extremity procedures, including modified Charles procedure, VLNT, and nodovenous shunt surgeries, demonstrated reductions in limb circumference and improvements in ambulation and daily functioning. Victor et al. reported limb circumference reductions exceeding 5 cm after debulking surgery, whereas Chilgar et al. demonstrated significant postoperative reductions in limb circumference at multiple levels following VLNT combined with excisional procedures [25,37]. Functional and psychological recovery were particularly emphasized among patients with advanced-stage disease. In contrast, minimally invasive or diagnostic procedures such as FNAC and aspiration mainly provided diagnostic clarification or temporary symptomatic relief rather than definitive disease control [12,13,14,15,16,17,18,19,20,21,22,23,24,25,26,27,28,29,30,31,32,33,34,35,36,37,38,39,40].

Complication profiles varied according to surgical technique and disease severity. Excisional and reconstructive procedures were primarily associated with wound-related complications, including superficial surgical site infection, serous discharge, edema, venous congestion, hypertrophic scarring, flap necrosis, cellulitis, and partial skin graft loss. The modified Charles procedure demonstrated the highest rate of postoperative hypertrophic scarring, reported in all patients, in addition to wound infection and ulceration. Microsurgical procedures such as VLNT and nodovenous shunts generally demonstrated favorable outcomes but remained associated with flap-related complications and seroma formation. Mortality and severe systemic deterioration were primarily observed in disseminated or progressive disease rather than as direct surgical complications. Overall, the comparative findings suggest that excisional debulking remains the most commonly utilized and effective intervention for advanced chronic filarial lymphedema, particularly in fibrotic disease, whereas physiologic procedures such as VLNT and nodovenous shunts may provide additional benefit in selected patients with preserved lymphatic function and less extensive tissue fibrosis [12,13,14,15,16,17,18,19,20,21,22,23,24,25,26,27,28,29,30,31,32,33,34,35,36,37,38,39,40].

### 3.4. Risk of Bias

The overall risk of bias across the included studies was generally low to moderate. All case reports assessed using the JBI checklist fulfilled all methodological domains, including clear reporting of patient demographics, clinical presentation, diagnostic methods, interventions, outcomes, adverse events, and key lessons learned, suggesting a consistently low risk of reporting bias among these studies. Similarly, the cohort studies evaluated using the Newcastle–Ottawa Scale (NOS) achieved high scores ranging from 6 to 7 stars, reflecting acceptable methodological quality with adequate selection, comparability, and outcome assessment domains overall. These findings support a reasonable level of confidence in the consistency of the reported clinical outcomes, although some methodological limitations remain present.

However, some concerns remain regarding the case series study assessed with the JBI checklist. Specifically, several domains were rated as “Unclear,” including inclusion criteria, completeness of participant inclusion, and reporting of participant demographics. These unclear items may indicate insufficient reporting transparency and introduce potential selection bias, as it is uncertain whether all eligible patients were included consecutively or whether important baseline characteristics were omitted.

Consequently, the representativeness and generalizability of the findings from this study may be limited. Although the overall body of evidence demonstrates acceptable methodological quality, the presence of unclear methodological domains reduces certainty in the pooled conclusions and should be considered when interpreting the findings. Therefore, conclusions drawn from this review should be interpreted with moderate caution, particularly regarding the external validity and reproducibility of outcomes across broader patient populations.

## 4. Discussion

The pathophysiology of lymphatic filariasis is fundamentally driven by the lodging of adult nematode worms within the lymphatic vessels, which triggers an intense local inflammatory response, endothelial dysfunction, and profound lymphatic dilation [41]. In the earlier phase, lymphedema caused thickening of the skin and structural changes in the subcutaneous tissue. As the disease progresses, the damage to the subcutaneous tissue also progresses; this might be due to greater fibrosis or fluid accumulation [42]. In the advanced stages of lymphedema targeted in this review, the lymphatic architecture undergoes irreversible fibrotic remodeling and sclerosis, severely compromising lymph flow and creating an immunologically compromised environment highly susceptible to acute dermatolymphangioadenitis (ADLA) attacks. Globally, it is estimated that over 15 million individuals suffer from filariasis-induced lymphedema, yet our systematic search revealed a striking discrepancy between modern lymphedema surgical paradigms and the realities of clinical management of filariasis [43]. While contemporary lymphedema treatment heavily emphasizes physiological reconstructions such as VLNT and lymphovenous anastomosis (LVA), our extracted data demonstrated an exclusive reliance on excisional debulking procedures. This divergence is likely rooted in the underlying pathology; by the time filarial patients reach the more advanced stage, the requisite lymphatic channels are functionally destroyed and physically obliterated by dense fibrosis, rendering delicate microvascular anastomoses anatomically unfeasible and necessitating ablative salvage techniques [44,45].

Furthermore, the clinical data highlighted a predominant focus on massive genitourinary involvement, specifically penoscrotal elephantiasis, rather than lower extremity lymphedema. The scrotal lymphatics serve as a common anatomic reservoir for adult filarial worms, leading to massive fluid accumulation, skin thickening, and, ultimately, superimposed fungal or bacterial infections [46]. Excisional debulking with complex genitourinary reconstruction, demonstrated high effectiveness for scrotal lymphedema [20,27,28,40,47]. By mechanically excising the fibrotic burden and removing the calcified remnants of dead worms that serve as a nidus for recurrent sepsis, patients experienced dramatic, life-altering improvements in mobility and psychosocial well-being [48]. Although historical perspectives often view excisional procedures as highly morbid, the studies synthesized in this review reported exceptionally low rates of severe surgical complications, with adverse events largely limited to manageable, superficial surgical site infections occurring in fewer than 10% of documented cases [49].

Despite these positive qualitative outcomes, the evidence base for the surgical management of filariasis remains critically underdeveloped compared with that for non-endemic, oncology-related lymphedema. The complete absence of comparative efficacy data in our findings underscores a significant failure of paradigm in tropical medicine research [50]. None of the twenty-nine included studies utilized conservative management as a defined control group, nor did any adequately quantify the primary outcome of percentage limb volume reduction using standardized objective metrics such as water displacement volumetry or bioimpedance spectroscopy. The literature is currently sustained by isolated case reports and small series that lack standardized longitudinal follow-up, highlighting an urgent clinical mandate to transition from anecdotal reporting to structured, comparative investigations that can rigorously define the optimal timing and modality of surgical intervention in advanced filarial disease.

Complete Decongestive Therapy

Complete Decongestive Therapy (CDT) remains a foundational component in the management of chronic lymphedema, even when definitive surgical treatment is being considered. CDT typically combines meticulous skin care, manual lymphatic drainage, compression bandaging or garments, and exercise, with the goal of reducing edema volume, softening fibrotic tissue, lowering bacterial and fungal colonization, and decreasing recurrent inflammatory attacks. In advanced filarial lymphedema, CDT alone may be insufficient to reverse established elephantiasis, but it can optimize the limb before surgery and help preserve postoperative gains afterward. Reports from the uploaded literature describe multimodal strategies in which CDT or related decongestive physical therapy was used preoperatively and postoperatively to improve tissue condition, facilitate wound healing, and reduce recurrence risk, supporting its role as an adjunct rather than a competing alternative to surgery [36,38,39].

However, the role of CDT in advanced filarial lymphedema appears largely supportive rather than curative [51]. Once patients progress to late-stage disease with dense fibrosis, marked skin thickening, and irreversible architectural destruction of the lymphatic system, conservative measures alone are often insufficient to restore meaningful drainage or reverse deformity [52]. This helps explain why many surgically treated patients in the present review had already reached a stage at which excisional procedures were used as salvage interventions. Accordingly, CDT should be viewed not as an alternative to surgery in all advanced cases, but as an essential component of perioperative optimization and long-term postoperative maintenance [53].

Physiologic and Reconstructive Surgical Procedures

Physiologic procedures aim to restore lymphatic drainage rather than simply remove diseased tissue. Lymphovenous anastomosis (LVA) diverts lymph from functioning lymphatic channels into nearby venules and is generally considered most effective when patent lymphatics remain. VLNT involves transfer of healthy lymph node–bearing tissue to the affected region to promote lymphangiogenesis and improve drainage, while nodovenous or lymphonodovenous shunts create an alternative pathway between lymphatic structures and the venous system. Although these approaches are well described conceptually, their application in filariasis-induced lymphedema remains limited and appears highly dependent on disease stage, residual lymphatic function, and local expertise. Available reports suggest that combining physiologic reconstruction with excisional debulking may offer better control of limb size, heaviness, and recurrent lymphangitis than debulking alone in selected patients, but robust comparative evidence is still lacking [35,36,37,38,39].

Comparison of Surgical Intervention to Conservative Management

Current evidence suggests that surgical intervention offers substantially greater functional and symptomatic improvement than conservative management alone in adults with filariasis-induced lymphedema, particularly in advanced-stage disease characterized by fibrosis, deformity, recurrent infection, and impaired mobility. Some cases of filariasis-induced lymphedema occurring in unusual anatomical locations as presented in this review, have also demonstrated that surgical intervention of mass excision, such as lumpectomy, cyst excision and mastectomy, can be a beneficial option in reducing lymphedema. Conservative measures—including complex decongestive therapy, compression, manual lymphatic drainage, antibiotics, hygiene optimization, and antiparasitic medications such as diethylcarbamazine or albendazole—remain important first-line approaches during early disease stages and for perioperative optimization. However, conservative treatment alone is often insufficient once irreversible lymphatic fibrosis and tissue hypertrophy develop. Patients undergoing excisional debulking procedures, modified Charles procedures, nodovenous shunts, or vascularized lymph node transfer (VLNT) consistently showed improved ambulation, reduction in limb circumference, enhanced hygiene, improved cosmesis, and marked psychosocial recovery compared with preoperative conservative management [25,37,39].

Advanced Diagnostic Imaging Modalities

Imaging is central to selecting appropriate candidates for physiologic lymphatic surgery and to understanding the severity of lymphatic dysfunction. Lymphoscintigraphy remains the most commonly reported functional imaging modality in the filariasis literature and can demonstrate delayed transport, dermal backflow, interrupted channels, and residual nodal function; these findings may help determine whether procedures such as nodovenous shunting or VLNT are feasible and can also be used for postoperative assessment. In contrast, indocyanine green (ICG) lymphography provides high-resolution real-time visualization of superficial lymphatic channels and is particularly useful for planning supermicrosurgical procedures such as LVA. However, ICG appears to be rarely reported in filariasis-specific surgical studies, likely reflecting limited availability in endemic settings and the fact that many patients present with advanced fibrosis, where superficial channels may already be severely damaged. As a result, lymphoscintigraphy currently remains more established in this field, while ICG represents a promising but underreported tool for future patient selection and operative planning [36,37].

Research Gaps and Future Directions

The current landscape of filarial lymphedema research reveals a critical paradigm failure, primarily due to the complete absence of comparative efficacy data. Currently, the literature is sustained by isolated case reports and small series that lack standardized longitudinal follow-up, making the evidence base highly susceptible to publication bias. None of the evaluated studies utilized defined conservative management as a control group, nor did they quantify postoperative volumetric reductions using standardized, objective tools such as bioimpedance spectroscopy or water displacement volumetry. None of the studies mention the evolution of subcutaneous changes post-surgical procedure. To establish definitive, evidence-based clinical guidelines, future research must urgently transition from anecdotal observational reporting to well-designed, prospective controlled trials that utilize standardized outcome metrics to clearly delineate the optimal timing, safety, and modality of surgical intervention [35,36,37,38,39].

Study Limitation

The findings of this systematic review must be interpreted within the context of several significant methodological limitations inherent to the primary literature. The overwhelming preponderance of single-patient case reports and small retrospective series introduces a high risk of publication bias, as successful or highly unusual clinical outcomes are disproportionately represented while surgical failures or modest improvements remain unpublished. Additionally, the complete lack of randomized controlled trials or prospective cohort studies with defined comparator groups entirely precludes the ability to perform a quantitative meta-analysis or establish definitive causality regarding surgical efficacy versus conservative medical management. Furthermore, the extracted literature demonstrated a profound heterogeneity in follow-up durations and a widespread failure to utilize validated, standardized assessment tools for quality of life or precise volumetric measurements, limiting the generalizability of the outcomes and preventing a standardized assessment of long-term surgical durability. The geographic distribution of included studies was heavily skewed toward India, with limited representation from other endemic regions such as sub-Saharan Africa and Southeast Asia, which may constrain external validity. Furthermore, the dataset was characterized by a pronounced imbalance between one large epidemiological study and numerous single-patient case reports, further limiting the robustness of pooled conclusions. The inclusion of studies with pediatric patients, despite the stated adult-focused inclusion criteria, represents an additional methodological inconsistency that should be considered when interpreting the findings.

## 5. Conclusions

In conclusion, surgical intervention remains an important therapeutic option for adult patients with advanced, medically refractory filariasis-induced lymphedema, particularly in cases associated with severe functional impairment. Current evidence suggests that excisional debulking procedures are commonly utilized in advanced-stage disease due to the extensive fibrosis and chronic lymphatic damage frequently observed in endemic filariasis. These procedures have been associated with symptomatic improvement, enhanced quality of life, and acceptable perioperative outcomes in several observational studies. However, the available evidence is limited predominantly to low-level, non-comparative studies, thereby precluding definitive conclusions regarding the relative effectiveness or superiority of specific surgical approaches. As physiological and microsurgical techniques continue to advance, a combination or staged approach of ablative and physiological surgery may prove useful to restore lymphatic flow, although their role in advanced filarial lymphedema remains insufficiently defined. Future well-designed prospective and controlled studies using standardized objective outcome measures are needed to clarify surgical indications, evaluate comparative effectiveness, optimize operative strategies, and guide evidence-based management for this debilitating disease.

## Figures and Tables

**Table 1:** Baseline Demographics and Study Characteristics.

Reference	Country	Study Design	Total Sample Size	Age	Sex Distribution	Mean Duration of Lymphedema Prior to Surgery	Baseline Lymphedema Stage	Prior Conservative Management	Anatomical Location
Gonzálvez-Gasch et al., 2025 [17]	Spain	Case Report	1	49 years	100% Male	Not reported	Not reported	Not reported	Face (subcutaneous)
Nunthanid et al., 2020 [24]	Thailand	Case Report	1	70 years	100% Female	Not reported	Not reported	Received dicloxacillin 250 mg four times a day for 2 weeks prior to surgery	Face/periorbital
Simmonds, 2018 [29]	USA	Case Report and Retrospective Database Analysis	1 case report + 865 database admissions	11 years (case report); 64.5 years (database average)	100% Female (case);	Not reported	Not reported	Not reported	Cervical (neck)
Panda, 2017 [26]	India	Case Report	1	28 years	100% Female	Not reported	Not reported	Not reported	Breast
Thongpiya, 2021 [32]	Thailand	Case Report	1	64 years	100% Female	Not reported	Not reported	Received anastrozole for two months before surgery to treat concomitant carcinoma	Breast
Tummidi, 2017 [33]	India	Case Report	1	35 years	100% Female	Not reported	Not reported	Not reported	Breast
Tandon et al., 2013 [30]	India	Case Report	2	Mean: 36.5 years	50% Male, 50% Female	Not reported	Not reported	Not reported	Upper extremity
Daggett et al., 2021 [14]	USA	Case Report	1	70 years	100% Male	5 years	Severe bilateral lymphedema with chronic wounds (WHO Grade 4)	Lasix prescribed as needed for edema, regular wound care, and empiric treatment with doxycycline and ivermectin was recommended	Upper and lower extremity
Gahine et al., 2019 [16]	India	Case Report	1	35 years	100% Male	Not reported	Not reported	Not reported	Hepatic/systemic
Jaiswal et al., 2013 [18]	India	Case Report	1	55 years	100% Male	Not reported	Not reported	Not reported	Adrenal/retroperitoneal
Diwakar et al., 2018 [15]	India	Case Report	1	52	100% Male	6 months (abdominal pain duration)	Not reported	Over-the-counter analgesics, antacids, and a proton pump inhibitor	Retroperitoneal
Connor et al., 2019 [13]	UK	Case Report	1	40	100% Male	Not specified	Not reported	Trial of steroids, bilateral Double-J stents, ivermectin, and doxycycline	Retroperitoneal
Kumar et al., 2023 [19]	India	Case Series	3	Mean: 37.3 years	100% Male	9.0 years	WHO Grade 4	Antibiotics and regular dressings utilized to control infection prior to surgical debulking	Genitourinary (scrotum/penis)
Mendu et al., 2017 [22]	USA	Case Reports	2	Mean: 73.0 years	100% Female	Not reported	Not reported	Not reported	Genitourinary (chyluria)
Sahu et al., 2020 [27]	India	Case Report	1	70	100% Male	30 years	Not reported	Not reported	Genitourinary (penis/scrotum)
Tekin et al., 2021 [31]	Denmark	Case Report	1	41	100% Male	3 years	Not reported	Extensive diagnostic workup (conservative therapies were noted as ineffective)	Genitourinary (scrotum)
Lema et al., 2025 [20]	Tanzania	Case Report	1	38	100% Male	28 years	Not reported	Sought assistance from traditional healers	Genitourinary (penoscrotal)
Saqlain et al., 2025 [28]	Pakistan	Case Report	1	17	100% Male	5 years	Not reported	Not reported	Genitourinary (scrotum)
Coseriu et al., 2022 [40]	Romania	Case Report	1	50	Male	32 years	Not reported	Prophylactic Intravenous broad-spectrum antibiotics	Genitourinary (penoscrotal)
Istranov et al., 2023 [39]	Russia	Case Report	1	48	Male	31 years	Not reported	Complex Decongestive Therapy.	Genitourinary (scrotum), lower extremity
Wijaya et al., 2016 [34]	Indonesia	Case Report	1	51	100% Male	17 years (scrotum), 5 years (left leg)	Not reported	Oral albendazole, diethylcarbamazine (DEC), captopril, sodium bicarbonate, folic acid, vitamin B12, and iron sucrose injection	Genitourinary (scrotum), lower extremity (bilateral legs)
Nayak et al., 2017 [23]	India	Case Report	1	60 years	100% Male	30 years	Not reported	Managed with dressing and oral antibiotics in another hospital	Lower extremity
Mangla et al., 2024 [21]	India	Case Report	1	69	100% Female	4 years	Not reported	Multiple filariasis treatments at a local hospital	Lower extremity
Victor et al., 2021 [25]	India	Retrospective study	16	Mean: 52.6 years	75% Male, 25% Female	Not reported	WHO Grade 4	Penicillin prophylaxis—Inj. Benzathine penicillin 1.2 million units intramuscular once every 3 weeks or tablet penicillin G 400 mg OD and daily foot care.	Lower extremity
Kasim et al., 2020 [38]	Indonesia	Case Report	1	28	Male	4 years		Albendazole therapy 1 *×* 400 mg, diethyl carbamazine 5 × 100 mg, cetirizine 2 *×* 10 mg, and vitamin B complex 2 × 1 tab.	Lower extremity
Chilgar et al., 2019 [37]	India	Retrospective study	17	Average 43 (±15, range 17–67).	52.9% Male, 47.1% Female	Not reported	29.4% ISL Grade 2, 70.6% ISL Grade 3	Not reported	Lower extremity
Sivaprakasam et al., 2018 [36]	India	Case Report	1	37	Female	27 years	WHO Grade 4	A course of oral Penicillin (800 mg/twice daily) for one week, Manual Lymph Drainage (MLD), Complete Decongestive Therapy (CDT), Respiratory physiotherapy and walking.	Lower extremity
Islam et al., 2022 [35]	Bangladesh	Prospective Study	11	45.45% in range 25–45; 54.55% in range 46–65	54.55% Male, 45.45% Female	Not reported	Advanced stage (Stage 3)	Not reported	Lower extremity
Azhar et al., 2025 [12]	India	Cross-sectional rapid epidemiological study	1226	Ranges: 67.6% were 15–59 years	54.2% Female, 45.8% Male	Not reported	19.3% had WHO Grade 3, 10.5% had WHO Grade 4	Not reported	Upper extremity, lower extremity, genitourinary (hydrocele)

Note: Baseline lymphedema staging was reported using different classification systems across studies. The eligibility criteria for this review used the WHO filariasis staging system (stages 3–6). Where individual studies reported staging using the International Society of Lymphology (ISL) grading system or other frameworks, the original classification is preserved as reported by the study authors.

**Table 2:** Surgical Interventions, Comparators, and Clinical Outcomes.

Reference	Surgical Indications	Surgical Approach	Comparator Group	Follow-Up Duration	Percentage Limb Volume Reduction	Secondary Outcomes (QoL, Cosmesis, Symptom Relief)	Complications or Adverse Events
Gonzálvez-Gasch et al., 2025 [17]	Painful subcutaneous facial swelling/cyst	Excisional (Complete excision of the cyst containing the worm)	Not reported	Not reported	Not reported	All symptoms resolved within 48 h after surgical removal	Not reported
Nunthanid et al., 2020 [24]	Erythematous periorbital nodule/infected thin-walled cyst	Excisional (Mass excision of the cyst)	Not reported	Not specified	Not reported	Surgical wound completely healed without secondary bacterial infection	None reported
Simmonds, 2018 [29]	Diagnosis of an enlarged, painless left cervical lymph node	Excisional biopsy of the left neck mass	Not reported	Patient did not present for follow-up care	Not reported	Not reported	Tolerated without complications.
Panda, 2017 [26]	Suspected breast neoplasm/organized breast abscess	Lumpectomy	Not reported	5 months post operatively (for recurrence); 6 weeks post-medical therapy	Not reported	Patient was symptomatically relieved and the lesion disappeared 6 weeks following subsequent medical therapy (diethylcarbamazine, amoxicillin + clavulanic acid, and doxycycline).	Occasional pain and recurrent nodularity 5 months post-surgery.
Thongpiya, 2021 [32]	Invasive ductal carcinoma (left breast); Filarial nodule (right breast) initially planned for removal	Left modified radical mastectomy; Ultrasound-guided core needle biopsy (right breast)	Not reported	2 months	Not reported	The right breast lesion resolved spontaneously without filarial medication.	Not reported
Tummidi, 2017 [33]	Diagnostic evaluation of a breast lump	Fine Needle Aspiration Cytology (FNAC)	Not reported	Not reported	Not reported	Not specified	None reported.
Tandon et al., 2013 [30]	Diagnostic Fine Needle Aspiration (FNA) only.	None reported.	None reported.	Not reported	Not reported	Swelling shrank in size following aspiration in Case.	None reported.
Daggett et al., 2021 [14]	Substernal goiter causing external compression of the thoracic duct and resulting in severe lymphedema	Left hemithyroidectomy via cervical incision and median sternotomy	Not reported	Approximately 6 months	Not reported	Patient regained mobility after being bed-bound for 5 years; quality of life greatly enhanced	No intraoperative or postoperative complications
Gahine et al., 2019 [16]	Not reported	Not reported	Not reported	Followed for 1 month	Not reported	Not reported	Died within 1 month due to complications and hepatic failure (disease progression)
Jaiswal et al., 2013 [18]	Not reported	Not reported	Not reported	Not reported	Not reported	Not reported	Not reported
Diwakar et al., 2018 [15]	Diagnostic uncertainty; failure of non-surgical medical therapies to relieve abdominal pain.	Excisional (Cyst excision).	Not reported	6 months.	Not reported	Disease-free with clear ultrasound.	Uneventful postoperative period.
Connor et al., 2019 [13]	Atypical retroperitoneal fibrosis causing hydronephrosis and renal failure.	Stent insertion, nephrostomy.	Not reported	Until death.	Not reported	No clinical improvement noted.	Multiorgan failure leading to death after repeated infections.
Kumar et al., 2023 [19]	Advanced disease stages where fibrosis has set in, causing severe physical limitations, disability, social stigma, and inability to maintain hygiene	Excisional debulking (reduction scrotoplasty) combined with reconstructive surgery of the external genitalia.	Not reported	3 months.	Not reported	Achieved acceptable/promising cosmetic results; alleviating psychosocial and physical distress; minimal to no recurrence.	Serous discharge requiring daily dressings; minor wound infection on postoperative day 5 requiring antibiotics; required subsequent revision surgery/grafting for penile coverage.
Mendu et al., 2017 [22]	Conservative management only: low fat, high fiber diet).	None reported.	None reported.	Several weeks to months.	None reported.	Spontaneous remission and disappearance of chyluria.	None reported.
Sahu et al., 2020 [27]	Discovery of a cauliflower-like squamous cell carcinoma growth under the prepuce of a ram’s horn penis.	Excisional debulking (Scrotectomy and total amputation of penis).	Not reported	2 weeks.	Not reported	Negative for inguinal metastasis; patient put on regular follow-up.	Uneventful postoperative recovery.
Tekin et al., 2021 [31]	Emergency admission due to grossly enlarged, hemorrhagic scrotum.	Excisional debulking (Subtotal scrotectomy and penoscrotal reconstruction).	Not reported	6 months.	Not reported	Complete wound closure, no lymphedema recurrence, and substantial improvement in QoL scores (ICIQ, SF-36).	Superficial surgical site infections (responded to antibiotics).
Lema et al., 2025 [20]	Chronic massive penoscrotal lymphedema complicated by an ulcer and secondary infection.	Excisional debulking (Urgent debridement, scrotoplasty, and penile skin grafting).	Not reported	6 months.	Not reported	Good functional and cosmetic outcomes maintained; satisfactory clearance of warts after adjunct medical therapy.	No complications observed postoperatively.
Saqlain et al., 2025 [28]	Discomfort, difficulty ambulating, impaired hygiene, and recurrent skin infections/ulcerations.	Excisional debulking (Subtotal scrotectomy with penile preservation).	Not reported	Not reported	Not reported	Significant relief from discomfort and improved mobility.	Uneventful postoperative recovery.
Coseriu et al., 2022 [40]	Massive penoscrotal edema	Ablative surgery, bilateral orchidopexy, abdominal advancement flaps, split-thickness skin graft.	Not reported	5 months post operatively	Not reported	Satisfactory esthetic and functional result.	Venous congestion of the flaps
Istranov et al., 2023 [39]	Repeated episodes of erysipelas and transient lymphorrhea, infertility.	Excision and Reconstructive plastic surgery: removal of the affected tissues of the urogenital region, phalloplasty, and scrotoplasty with rotation skin flaps	Not reported	3 months post operatively	Right lower limb: 10.5 cm; Left lower limb: 6.0 cm; Scrotum: 62 cm	Significant emotional recovery, along with functional (activities and sexual) recovery.	Edema
Wijaya et al., 2016 [34]	Giant recurrent scrotal swelling and bilateral leg swelling.	Excisional debulking (Scrotal reconstruction surgery).	Not reported	Not reported	Not reported	Not reported	Not reported
Nayak et al., 2017 [23]	Foul-smelling multiple ulcero-proliferative lesions/amelanotic melanoma on a filarial leg	Excisional (Wide local excision of the tumor followed by skin grafting)	Not reported	9 months post operatively until brain metastasis developed; 3 months post-brain surgery	Not reported	Symptomatic improvement after the subsequent brain surgery	Developed right-sided hemiparesis and brain metastasis after 9 months (disease progression, not a direct surgical complication)
Mangla et al., 2024 [21]	Non-healing painful ulcer diagnosed as squamous cell carcinoma.	Excisional debulking (Wide local excision with primary closure).	Not reported	Ongoing.	Not reported	Patient was started on immunotherapy for nodal metastasis.	No direct surgical complications reported; progression of swelling noted.
Victor et al., 2021 [25]	WHO Grade 4	Nodovenous shunt + debulking surgery = 14. Nodovenous shunt + sculpting surgery = 2.	Not reported	1 to 3 years	Nodovenous shunt surgery: Limb circumference reduction of 2.6 cm; Debulking surgery: Limb circumference reduction of > 5 cm	Not reported	Skin flap necrosis in three patients, seroma at the site of the nodovenous shunt in one patient, and cellulitis in two patients.
Kasim et al., 2020 [38]	Fever, pain, swollen legs	Removal of fibrotic tissue and edematous subcutaneous tissue	Not reported	2 years postoperatively	Not reported	Satisfactory leg size reduction	None reported
Chilgar et al., 2019 [37]	Not reported	Vascularized lymph node transfer (VLNT) along with excisional procedure	Not reported	3 to 16 months postoperatively	Significant reduction in leg circumference at Above knee, Below knee, Above ankle, Below ankle (*p* < 0.005)	Satisfactory improvement of limb size and functional outcome, significant reduction in feeling of heaviness and episodes of acute lymphangitis after surgery, significant increase in level of self-confidence after surgery.	Donor site seroma was present only in one case. In six patients, the skin graft on flap site was partially lost.
Sivaprakasam et al., 2018 [36]	Not reported	Supra-fascial excision of alternate lumps in three stages at an interval of 6 weeks, followed by a nodo-venal shunt	Not reported	10 years	Not reported	Not reported	Not reported
Islam et al., 2022 [35]	Discomfort, infections, ulceration, nodularity, warty lesion, loss of movement, and the inability to wear conventional clothing and shoes	Modified Charles Procedure	Not reported	5.6 months	Not reported	Continuation of normal function and ambulation, and considerable improvements in quality of life, recovery of depressive illness.	Wound infection in 18.18%, Ulceration in 18.18%, Hypertrophic scar in 100% patients.
Azhar et al., 2025 [12]	Not reported	Not reported	Not reported	Not reported	Not reported	Not reported	After mass drug administration (not surgery), 2% reported side effects like headache, nausea, and weakness.

## Data Availability

The original data presented in the study are openly available in https://doi.org/10.5281/zenodo.20110806.

## Abbreviations

The following abbreviations are used in this manuscript:

ADLA	Acute dermatolymphangioadenitis
CDT	Complete Decongestive Therapy
DEC	Diethylcarbamazine
FNA	Fine Needle Aspiration
FNAC	Fine Needle Aspiration Cytology
ICG	Indocyanine green
ISL	International Society of Lymphology
JBI	Joanna Briggs Institute
LVA	Lymphovenous Anastomosis
MLD	Manual Lymph Drainage
NOS	Newcastle-Ottawa Scale
PICO	Population, Intervention, Comparison, and Outcomes
PRISMA	Preferred Reporting Items for Systematic Reviews and Meta-Analyses
VLNT	Vascularized Lymph Node Transfer
WHO	World Health Organization
